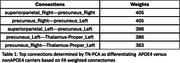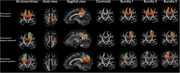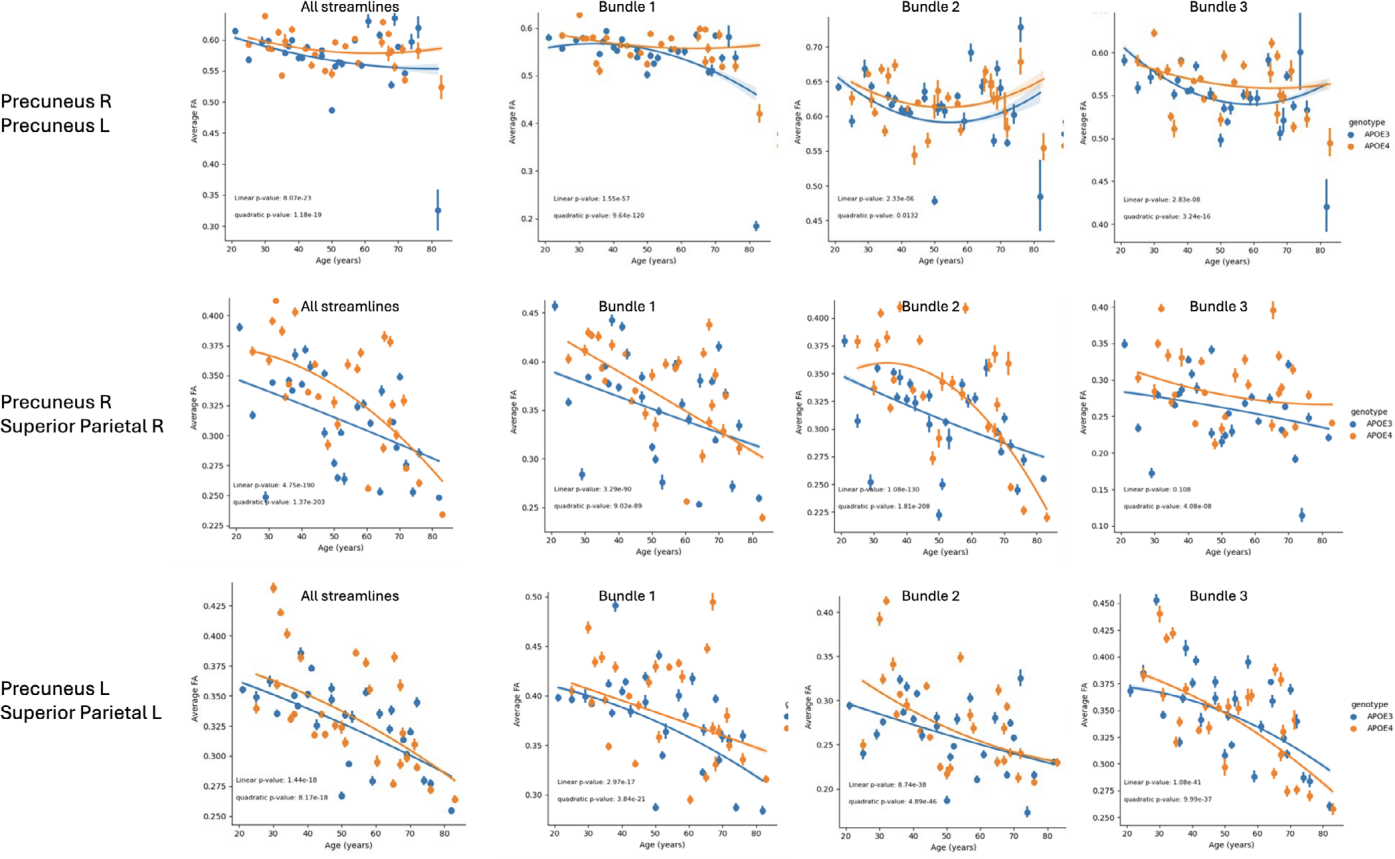# Connectomic differences in cognitively normal APOE4 versus non‐APOE4 carriers at risk for Alzheimer’s Disease

**DOI:** 10.1002/alz.092992

**Published:** 2025-01-09

**Authors:** Jacques Andrew Stout, Ali Mahzarnia, Hae Sol Moon, Zay Yar Han, Kim G Johnson, Alexandra Badea

**Affiliations:** ^1^ Brain Imaging and Analysis Center, Duke University Medical Center, Durham, NC USA; ^2^ Duke University, Durham, NC USA; ^3^ Radiology Department, Duke University Medical Center, Durham, NC USA; ^4^ Biomedical Engineering Department, Duke University, Durham, NC USA; ^5^ Neurology Department, Duke University Medical Center, Durham, NC USA

## Abstract

**Background:**

Alzheimer's disease (AD) causes a steady degradation of connections inside the brain. The apolipoprotein E is a protein where one of its subtypes, APOE4, is a major genetic risk factor for developing late onset AD. Using a combination of tensor network PCA (TN‐PCA) and bundle analysis, we sought to determine which specific connections differentiate APOE4 individuals relative to non‐APOE4 carriers, and whether these changes increase with age.

**Method:**

Our study included 77 individuals from 20 to 83 years old, 37 male and 40 female, 41 possessing only the APOE3 allele and 36 with at least one APOE4 allele. Imaging was done using diffusion weighted imaging, with a T1w anatomical scan also obtained for atlas based labeling and co‐registration. The tracts were modeled in subject space using mrtrix with anatomically constrained tractography and brought into the same template image space using our SAMBA registration pipeline. Connectomes of number of streamlines and the average FA were calculated with mrtrix. FA connectomes were used with TN‐PCA, determining which connections had higher weight for separating the genotype groups between APOE3 and APOE4. Streamlines of specific connections were split into bundles using dipy quickbundles. Statistical analysis compared linear and quadratic models for age, genotype and the interaction of the two.

**Result:**

TN‐PCA results indicate that connections of precuneus to other structures had an important weight in APOE3 to APOE4 differentiation, particularly their inter‐hemispheric connection, their connection to the superior‐parietal region. Linear modeling showed the FA of precuneus connected streamlines had noticeable differences between APOE3 and APOE4 groups. These differences were exacerbated in specific bundles: precuneus‐right to precuneus‐left, and precuneus‐left to superiorparietal‐left connection.

**Conclusion:**

Our results indicate that the precuneus connections differentiate between APOE4 and non‐APOE4 carriers. Future studies will address separately the white matter and grey matter streamline sections, and we will add further analysis along the streamlines for increased specificity.